# On the Stability of Disubstituted Cyclobutenes – A Computational Study

**DOI:** 10.1002/ejoc.201801243

**Published:** 2018-11-21

**Authors:** Boris Maryasin, Nuno Maulide

**Affiliations:** ^1^ Institute of Organic Chemistry University of Vienna Währinger Strasse 38 1090 Vienna Austria; ^2^ Institute of Theoretical Chemistry University of Vienna Währinger Strasse 17 1090 Vienna Austria

**Keywords:** Cyclobutenes, Ring‐opening, Density functional calculations, Reaction mechanisms, Substituent effects

## Abstract

A computational study of the electrocyclic ring‐opening of 2‐substituted cyclobutenecarboxylic acids is presented. Detailed calculations suggest a model to predict whether the product of nucleophilic alkylation of a bicyclic lactone electrophile will be a cyclobutenecarboxylic acid or its dienoic acid isomer, based on the used nucleophile.

## Introduction

**Scheme 1:**
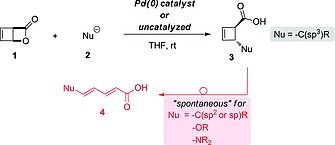
Transformation of bicyclic lactone **1** to cyclobutenes **3** and their electrocyclic ring opening rearrangements to dienoic acids **4**.

Our group has developed a research program centered on palladium‐catalyzed and uncatalyzed ring‐opening reactions of bicyclic lactone **1** with a broad range of nucleophiles (Scheme 1, top).^1^, ^2^, ^3^, ^4^, ^5^ These transformations constitute a platform for access to functionalized cyclobutenes with high and unusual diastereo‐ and enantioselectivities.

It has been experimentally observed that the reaction outcome critically depends on the nature of the nucleophile **2**. In the case of nitrogen‐ or oxygen‐centered species, as well as for acetylenic,^6^ vinylic or aromatic carbon‐centered nucleophiles, a spontaneous electrocyclic ring opening **3**→**4** (Scheme 1, bottom) takes place at close to room temperature. This leads to the dienoic acid **4** 7, 8 and the cyclobutene **3** cannot be isolated. In contrast, most sp^3^‐hybridised carbon nucleophiles generate cyclobutenes **3** from which ring‐opening to the diene **4** can be triggered, but only under elevated temperatures.

**Scheme 2:**
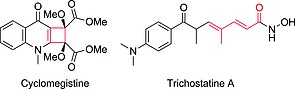
Examples of natural products containing cyclobutene and dienoic moieties.

Both the cyclobutenes **3** and their isomeric dienes **4** are attractive synthetic targets and common fragments of important biologically active natural products. Two examples are presented in Scheme 2, and we have previously explored the torquoselective ring‐opening of stereodefined cyclobutenes for the preparation of valuable building blocks or natural products.^6^, ^7^, ^9^ Thus, it is desirable to understand the factors controlling the divergent selectivity of the reactions displayed in Scheme 1.

Here we present a computational study aimed at answering the question: what is the correlation between the propensity towards undergoing the electrocyclic ring‐opening **3**→**4** at or close to room temperature and the structural features of nucleophile **2**?

## Results and Discussion

**Scheme 3:**
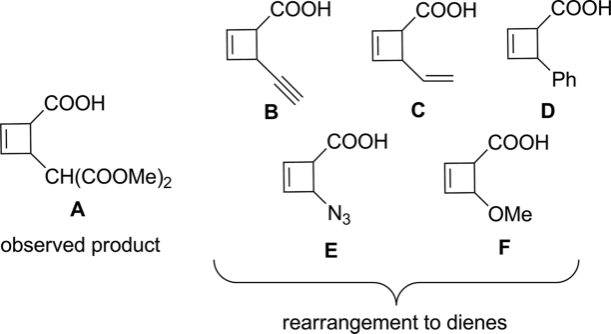
Six cyclobutene derivatives **A**–**F** (*cis* and *trans*) chosen for the computational study.

The outcome of the transformation shown in Scheme 1 depends naturally on the kinetic and(or) thermodynamic stability of the cyclobutene derivative **3**. This stability is determined by molecular structure. Therefore, for the calculations we have chosen six characteristic cyclobutenes (both *cis* and *trans*) with different substituents R (Scheme 3). Our goals were: a) to test whether the kinetic and the thermodynamic features of the rearrangement **3**→**4** for these six systems agree with experimental observations; and b) to understand which factors impart stabilizing/destabilizing effects for the reaction **3**→**4**. The system **A** [R = CH(COOMe)_2_] is assumed to be stable as a cyclobutene derivative at room temperature accordingly to experimental data.^1^ In contrast, for the systems **B** (R = C≡CH), **C** (R = CH=CH_2_), **D** (R = Ph), **E** (R = N_3_) and **F** (R = OMe) “spontaneous” electrocyclic ring‐opening to dienes was observed.^6^, ^7^, ^8^

**Scheme 4:**
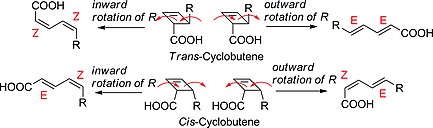
Torquoselectivity modes for the conrotatory electrocyclic ring‐opening of 2,3‐disubstituted cyclobutenes.

It has been shown that the thermally allowed, conrotatory 4π‐electrocyclic ring‐opening of cyclobutenes to butadienes^10^ can proceed via two pathways – namely, inward and outward rotation – leading to (*Z,Z*) and (*E,E*) (in case of *trans*‐cyclobutenes) or (*Z,E*) and (*E,Z*) (*cis*‐cyclobutenes) dienes respectively (Scheme 4). It has been shown that the preferred pathway depends on the nature of substituents.^11^, ^12^, ^13^, ^14^ Our results suggest a preference for outward rotation of the investigated *trans*‐ and *cis*‐cyclobutenes (Table S3 of the Supporting Information), which is in accordance with the torquoselectivity model elaborated by Houk and Niwayama for the 4π‐electrocyclic ring‐opening of cyclobutenes.^12^, ^13^

**Figure 1:**
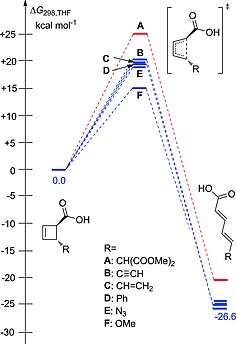
Free energy profile at B3LYP‐D3/def2‐TZVP level of theory for the reaction **3**(*trans*)→**4**(*E,E*). The *trans*‐cyclobutene is taken as a reference (0.0 kcal mol^–1^). Red colour for the system **A** highlights the high kinetic barrier for the ring‐opening event.

Table 1. and Figure 1 depict the Gibbs free energy profiles for the ring‐opening transformations of the considered systems **A**–**F**. In good agreement with the experimental data, the activation barrier for substrate **A** (25.5 kcal mol^–1^) is too high for a room temperature reaction, while the barriers for the systems **B**–**F** are substantially smaller (21.0, 19.9, 19.1, 18.8, 15.1 kcal mol^–1^ respectively) and the reaction **3**→**4** for those systems **B**–**F** is expected to proceed spontaneously. The relatively high barrier for the system **B** (21.0 kcal mol^–1^) agrees well with the experimentally observed substantial time (12 h) needed for the respective ring‐opening of a similar system to reach completion at room temperature.^6^ Table S3 of the Supporting Information presents the half‐lives obtained using the computed barriers and the Eyring equation. For the *trans*‐cyclobutene **A** the estimated half‐life is 6.5 days, while in the case of **F** the ring‐opening is much faster, and the half‐life is approx. 0.01 seconds. Table 1 demonstrates that the investigated *trans*‐ and *cis*‐cyclobutenes show similar kinetic and thermodynamic behavior, namely the rate of the ring‐opening reaction is decreasing from the **A** to the **F**.

**Table 1:** Relative Gibbs free energies (in kcal mol^–1^) for stationary points located on the potential‐energy surface for *trans*‐ and *cis*‐cyclobutene derivatives **A**–**F**. The cyclobutene is taken as a reference (0.0 kcal mol^–1^)

	*trans*‐cyclobutene			*cis*‐cyclobutene		
	CB^a^	TS	DI(*E,E*)	CB^b^	TS	DI(*Z,E*)
**A**	0.0	25.5	–19.7	0.0(1.0)	25.7	–18.9
**B**	0.0	21.0	–24.8	0.0(0.4)	21.9	–23.0
**C**	0.0	19.9	–26.6	0.0(0.5)	19.7	–25.2
**D**	0.0	19.1	–24.8	0.0(0.9)	18.5	–23.9
**E**	0.0	18.8	–24.4	0.0(1.2)	17.2	–23.8
**F**	0.0	15.1	–24.9	0.0(1.5)	14.7	–24.9

a CB: cyclobutene, TS: transition state, DI: dienoic product.

b The values in the brackets are the Gibbs free energies of *cis*‐cyclobutenes relatively to *trans*‐cyclobutenes.

**Figure 2:**
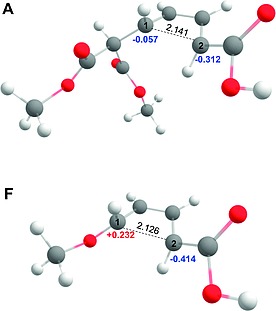
Structures of the transition states (outward, *trans*‐cyclobutene) for systems **A** and **F**. NBO charges on the atoms C1 and C2 are indicated (u.a.) and the bond lengths for the breaking bond C1–C2 is depicted [Å].

The ring‐opening of the considered cyclobutenes leads to dienes, the thermodynamic stability of which is not identical. Figure 1 and Table 1 show that dienes resulting from opening of **B**‐**F** are ≈ 5 kcal mol^–1^ more stable as compared to the **A** case. This is the result of a stabilizing interaction between the conjugated diene systems and the orbitals of the adjacent atoms of the substituent leading to what are effectively “push‐pull“ systems.^15^, ^16^, ^17^ These results are also in agreement with previous computational studies of disubstituted cyclobutenes.^18^ Thus, both kinetic and thermodynamic controls work in tandem [the slower reactions (e.g. **A**) lead to the thermodynamically less stable product as compared to the faster reactions (e.g. **F**)], and the highest transition state **A** is “later“ as compared to the lowest transition state **F**. This is also seen from the structures of these transition states presented in Figure 2 – the breaking bond C1–C2 is 0.015 Å longer (therefore “closer” to the “open product“ diene) in the transition state **A**.

**Figure 3:**
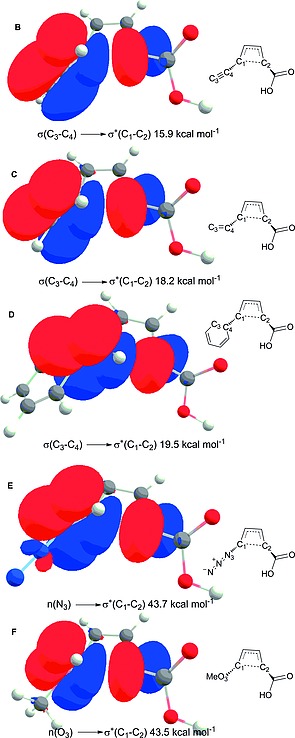
NBO orbitals: donor acceptor interaction stabilizing transition states **B**–**D**.

Natural Bond Orbitals^19^ (NBO) analysis of the transition state **F** (the smallest barrier) suggests that there is a donor–acceptor interaction from the lone electron pair (p orbital) on the oxygen atom of the methoxy group to the neighboring antibonding orbital of the C1–C2 bond. The energy of this interaction is computed to be 43.5 kcal mol^–1^ (Figure 3). Analogously, electron donation from the lone pair at nitrogen atom in case of the system **E** amounts to 43.7 kcal mol^–1^. Interestingly, the bonding orbitals of the corresponding alkynyl‐, vinyl‐ and phenyl‐substituted products (**B**–**D**) display a similar interaction with the C1–C2 antibonding orbital. In strong contrast, within the transition state **A** no stabilizing donor/acceptor interaction is present, which agrees with the high activation barrier predicted by our calculations (Figure 1). Noteworthy the values of the stabilizing donor–acceptor interaction correlate with the computed barriers, e.g. for the system **B** (the highest barrier among “kinetically allowed” ring‐opening reactions) the donor–acceptor energy is the smallest one (15.9 kcal mol^–1^ vs. ≈ 44 kcal mol^–1^ for the systems **E** and **F**).

## Conclusions

In this work, we have analyzed and interpreted the stability of a range of representative cyclobutenecarboxylic acids towards 4π‐electrocyclic ring opening. Taken together, our results rationalise the experimental evidence. Cyclobutenes synthesized from the bicyclic lactone **1** (Scheme 1) rearrange to the corresponding diene derivatives, if the substituent adjacent to the carboxylic group is able to electronically stabilize the resulting product and the respective transition state of the ring‐opening event. Stabilization of the transition states plays the crucial role of kinetically allowing or forbidding the reactions at room temperature. However, all investigated ring‐opening processes are found to be highly exergonic (–20 to –25 kcal mol^–1^). Thus, the ring‐opening transformation takes place for (in increasing order of propensity) alkynyl, vinyl, and phenyl derivatives, azido‐ or methoxy‐substituted cyclobutenes. If the substituent is connected to the cyclobutene by a sp^3^‐configured carbon atom, the rearrangement has a higher kinetic barrier, preventing the ring‐opening reaction from taking place at room temperature.

### Computational Details

The conformational space of all flexible molecules has been initially searched using OPLS_2005 force field^20^ and the systematic Monte Carlo conformers search routine implemented in MACROMODEL 11.5.^21^ The structures located at force field level have then been reoptimized at the B3LYP‐D3/def2‐TZVP^22^, ^23^, ^24^, ^25^, ^26^, ^27^, ^28^ level of theory. The nature of all stationary points (minima and transition states) was verified through computation of the vibrational frequencies. The thermal corrections to the Gibbs free energy were combined with the energies obtained at the same level of theory to yield DFT Gibbs free energies (*G*_298_) at 298.15 K. The density‐based solvation model SMD^29^ was applied to consider solvent (THF) effects. All energies are reported in kcal mol^–1^. The DFT calculations have been performed with the Gaussian09 program package.^30^ Computed structures were visualized using the Chemcraft software.^31^

## Supporting information

Supporting Information
