# Supplementary material for: On the Stability of Disubstituted Cyclobutenes – A Computational Study
Source: European J Org Chem. 2018 Nov 21;2019(2-3):338–41. doi: 10.1002/ejoc.201801243 (PMC6472590; doi:10.1002/ejoc.201801243)
Supplement: Supplementary file 1 — Supporting Information [file EJOC-2019-338-s001.pdf]

Supporting Information

On the Stability of Disubstituted Cyclobutenes – A Computational Study

Boris Maryasin* and Nuno Maulide*

ejoc201801243-sup-0001-SupMat.pdf

Table of Contents

Total energies and free energies of reactants, products and transition states 1

Structural parameters of the investigated cyclobutenes 11

Outward vs inward rotation and calculated half-lives 11

Cartesian coordinates for the most stable ($\Delta G_{298,DCM}$) conformations as computed at the B3LYP-D3-SMD/def2-TZVP level of theory 13

Total energies and free energies of reactants, products and transition states

Table S1. Total energies (Hartree), Gibbs free energies (Hartree), relative free energies (kj mol^{-1})

System	E_{tot}	G_{corr}	G	G_{rel}
A_CB_trans_1	-839.997845011	0.168017	-839.829828011	0.0
A_CB_trans_2	-839.997771205	0.168475	-839.829296205	1.4
A_CB_trans_3	-839.998072695	0.168961	-839.829111695	1.9
A_CB_trans_4	-839.998072677	0.168965	-839.829107677	1.9
A_CB_trans_5	-839.998361947	0.169343	-839.829018947	2.1
A_CB_trans_6	-839.997752497	0.168744	-839.829008497	2.2
A_CB_trans_7	-839.997169995	0.168338	-839.828831995	2.6
A_CB_trans_8	-839.997604361	0.168913	-839.828691361	3.0
A_CB_trans_9	-839.997463138	0.16888	-839.828583138	3.3
A_CB_trans_10	-839.997788221	0.169223	-839.828565221	3.3
A_CB_trans_11	-839.997169894	0.168633	-839.828536894	3.4
A_CB_trans_12	-839.997169899	0.168667	-839.828502899	3.5
A_CB_trans_13	-839.996546362	0.168199	-839.828347362	3.9
A_CB_trans_14	-839.996546181	0.168214	-839.828332181	3.9
A_CB_trans_15	-839.998434247	0.170111	-839.828323247	4.0
A_CB_trans_16	-839.99843444	0.170122	-839.82831244	4.0
A_CB_trans_17	-839.997790625	0.169751	-839.828039625	4.7
A_CB_trans_18	-839.99779063	0.169775	-839.82801563	4.8
A_CB_trans_19	-839.997526213	0.169515	-839.828011213	4.8
A_CB_trans_20	-839.997526213	0.169518	-839.828008213	4.8
A_CB_trans_21	-839.997141336	0.169233	-839.827908336	5.0
A_CB_trans_22	-839.996607554	0.168707	-839.827900554	5.1
A_CB_trans_23	-839.996541156	0.168681	-839.827860156	5.2
A_CB_trans_24	-839.996840863	0.169164	-839.827676863	5.6
A_CB_trans_25	-839.996840858	0.169164	-839.827676858	5.6
A_CB_trans_26	-839.996062322	0.16842	-839.827642322	5.7
A_CB_trans_27	-839.996464922	0.168891	-839.827573922	5.9
A_CB_trans_28	-839.997105469	0.169709	-839.827396469	6.4
A_CB_trans_29	-839.996791109	0.169447	-839.827344109	6.5
A_CB_trans_30	-839.997159689	0.169832	-839.827327689	6.6
A_CB_trans_31	-839.996433871	0.169354	-839.827079871	7.2
A_CB_trans_32	-839.995069393	0.168023	-839.827046393	7.3

A_CB_trans_33	-839.995970569	0.168944	-839.827026569	7.4
A_CB_trans_34	-839.995970584	0.168953	-839.827017584	7.4
A_CB_trans_35	-839.995691449	0.168701	-839.826990449	7.5
A_CB_trans_36	-839.995691446	0.168701	-839.826990446	7.5
A_CB_trans_37	-839.996396959	0.169576	-839.826820959	7.9
A_CB_trans_38	-839.995974022	0.169225	-839.826749022	8.1
A_CB_trans_39	-839.995301696	0.168594	-839.826707696	8.2
A_CB_trans_40	-839.995301696	0.168594	-839.826707696	8.2
A_CB_trans_41	-839.994870758	0.168196	-839.826674758	8.3
A_CB_trans_42	-839.996527286	0.169918	-839.826609286	8.5
A_CB_trans_43	-839.995324681	0.168905	-839.826419681	8.9
A_CB_trans_44	-839.996691408	0.170372	-839.826319408	9.2
A_CB_trans_45	-839.994825453	0.168567	-839.826258453	9.4
A_CB_trans_46	-839.995788442	0.169603	-839.826185442	9.6
A_CB_trans_47	-839.995584136	0.169468	-839.826116136	9.7
A_CB_trans_48	-839.995469099	0.169417	-839.826052099	9.9
A_CB_trans_49	-839.995469018	0.169434	-839.826035018	10.0
A_CB_trans_50	-839.99496079	0.169038	-839.82592279	10.3
A_CB_trans_51	-839.995311591	0.16944	-839.825871591	10.4
A_CB_trans_52	-839.994773726	0.169526	-839.825247726	12.0
A_CB_trans_53	-839.994600797	0.16971	-839.824890797	13.0
A_CB_trans_54	-839.994241569	0.169628	-839.824613569	13.7
A_CB_trans_55	-839.993845379	0.169267	-839.824578379	13.8
A_CB_trans_56	-839.993774671	0.169846	-839.823928671	15.5
A_CB_trans_57	-839.994110767	0.170818	-839.823292767	17.2
A_TS_trans_1	-839.954844344	0.165716	-839.789128344	0.0
A_TS_trans_2	-839.954835102	0.165919	-839.788916102	0.6
A_TS_trans_3	-839.9548351	0.165919	-839.7889161	0.6
A_TS_trans_4	-839.95481718	0.166071	-839.78874618	1.0
A_TS_trans_5	-839.954817168	0.166071	-839.788746168	1.0
A_TS_trans_6	-839.95393387	0.165229	-839.78870487	1.1
A_TS_trans_7	-839.954001166	0.165595	-839.788406166	1.9
A_TS_trans_8	-839.954001373	0.165685	-839.788316373	2.1
A_TS_trans_9	-839.952877645	0.164582	-839.788295645	2.2
A_TS_trans_10	-839.952624933	0.164334	-839.788290933	2.2
A_TS_trans_11	-839.954001632	0.165732	-839.788269632	2.3
A_TS_trans_12	-839.952878013	0.164779	-839.788099013	2.7
A_TS_trans_13	-839.954797786	0.166757	-839.788040786	2.9
A_TS_trans_14	-839.954797786	0.166757	-839.788040786	2.9
A_TS_trans_15	-839.954797786	0.166757	-839.788040786	2.9
A_TS_trans_16	-839.953936167	0.165896	-839.788040167	2.9
A_TS_trans_17	-839.954797744	0.166767	-839.788030744	2.9
A_TS_trans_18	-839.953178837	0.16515	-839.788028837	2.9
A_TS_trans_19	-839.952718499	0.164712	-839.788006499	2.9
A_TS_trans_20	-839.953171719	0.16519	-839.787981719	3.0
A_TS_trans_21	-839.953171353	0.16523	-839.787941353	3.1

A_TS_trans_22	-839.953879083	0.165974	-839.787905083	3.2
A_TS_trans_23	-839.953742862	0.165874	-839.787868862	3.3
A_TS_trans_24	-839.953742861	0.165874	-839.787868861	3.3
A_TS_trans_25	-839.954729481	0.167045	-839.787684481	3.8
A_TS_trans_26	-839.953988937	0.166317	-839.787671937	3.8
A_TS_trans_27	-839.953988763	0.166352	-839.787636763	3.9
A_TS_trans_28	-839.953988717	0.166356	-839.787632717	3.9
A_TS_trans_29	-839.953963491	0.166635	-839.787328491	4.7
A_TS_trans_30	-839.953963462	0.166637	-839.787326462	4.7
A_TS_trans_31	-839.952998829	0.165677	-839.787321829	4.7
A_TS_trans_32	-839.952998865	0.165685	-839.787313865	4.8
A_TS_trans_33	-839.953697187	0.166436	-839.787261187	4.9
A_TS_trans_34	-839.952839061	0.165617	-839.787222061	5.0
A_TS_trans_35	-839.952301507	0.165269	-839.787032507	5.5
A_TS_trans_36	-839.952301495	0.165283	-839.787018495	5.5
A_TS_trans_37	-839.953690572	0.166683	-839.787007572	5.6
A_TS_trans_38	-839.953088473	0.166089	-839.786999473	5.6
A_TS_trans_39	-839.953740508	0.166803	-839.786937508	5.8
A_TS_trans_40	-839.953740515	0.166805	-839.786935515	5.8
A_TS_trans_41	-839.953740478	0.166809	-839.786931478	5.8
A_TS_trans_42	-839.952204746	0.165422	-839.786782746	6.2
A_TS_trans_43	-839.952204732	0.165422	-839.786782732	6.2
A_TS_trans_44	-839.953054797	0.166419	-839.786635797	6.5
A_TS_trans_45	-839.953081328	0.166503	-839.786578328	6.7
A_TS_trans_46	-839.953081286	0.16651	-839.786571286	6.7
A_TS_trans_47	-839.95308127	0.166513	-839.78656827	6.7
A_TS_trans_48	-839.952414571	0.16593	-839.786484571	6.9
A_TS_trans_49	-839.952555315	0.16638	-839.786175315	7.8
A_TS_trans_50	-839.952555382	0.166386	-839.786169382	7.8
A_TS_trans_51	-839.952555387	0.166387	-839.786168387	7.8
A_TS_trans_52	-839.951947732	0.166116	-839.785831732	8.7
A_TS_trans_53	-839.95243143	0.166666	-839.78576543	8.8
A_TS_trans_54	-839.952431424	0.166666	-839.785765424	8.8
A_TS_trans_55	-839.952222133	0.166483	-839.785739133	8.9
A_TS_trans_56	-839.952869909	0.167324	-839.785545909	9.4
A_TS_trans_57	-839.952869892	0.167326	-839.785543892	9.4
A_TS_trans_58	-839.953690573	0.166683	-839.787007573	5.6
A_TS_trans_59	-839.953690571	0.166683	-839.787007571	5.6
A_TS_trans_inward_1	-839.944731857	0.167368	-839.777363857	30.9
A_TS_trans_inward_2	-839.944732081	0.167369	-839.777363081	30.9
A_TS_trans_inward_3	-839.944688419	0.167478	-839.777210419	31.3
A_TS_trans_inward_4	-839.94468814	0.167497	-839.77719114	31.3
A_TS_trans_inward_5	-839.944958294	0.168597	-839.776361294	33.5
A_TS_trans_inward_6	-839.944958217	0.168603	-839.776355217	33.5
A_TS_trans_inward_7	-839.944958217	0.168603	-839.776355217	33.5
A_TS_trans_inward_8	-839.944958214	0.168603	-839.776355214	33.5

A_TS_trans_inward_9	-839.944910037	0.168752	-839.776158037	34.1
A_TS_trans_inward_10	-839.944910032	0.168752	-839.776158032	34.1
A_TS_trans_inward_11	-839.943653737	0.167791	-839.775862737	34.8
A_TS_trans_inward_12	-839.943899737	0.168252	-839.775647737	35.4
A_TS_trans_inward_13	-839.943899719	0.168262	-839.775637719	35.4
A_TS_trans_inward_14	-839.943899707	0.168277	-839.775622707	35.5
A_TS_trans_inward_15	-839.938365157	0.167612	-839.770753157	48.2
A_DI_EE_1	-840.028432658	0.167153	-839.861279658	0.0
A_DI_EE_2	-840.028124452	0.166878	-839.861246452	0.1
A_DI_EE_3	-840.028124512	0.166884	-839.861240512	0.1
A_DI_EE_4	-840.027623246	0.166424	-839.861199246	0.2
A_DI_EE_5	-840.027623207	0.166429	-839.861194207	0.2
A_DI_EE_6	-840.027858299	0.167289	-839.860569299	1.9
A_DI_EE_7	-840.027315723	0.167229	-839.860086723	3.1
A_DI_EE_8	-840.026651294	0.16691	-839.859741294	4.0
A_DI_EE_9	-840.027194653	0.167528	-839.859666653	4.2
A_DI_EE_10	-840.026544351	0.167215	-839.859329351	5.1
A_DI_EE_11	-840.025005622	0.166193	-839.858812622	6.5
A_DI_EE_12	-840.025005571	0.166207	-839.858798571	6.5
A_DI_EE_13	-840.025367333	0.167452	-839.857915333	8.8
A_DI_EE_14	-840.024788113	0.166941	-839.857847113	9.0
A_DI_EE_15	-840.025416564	0.167809	-839.857607564	9.6
A_DI_EE_16	-840.022639596	0.165165	-839.857474596	10.0
A_DI_EE_17	-840.024971246	0.167611	-839.857360246	10.3
A_DI_EE_18	-840.024756096	0.167924	-839.856832096	11.7
A_DI_EE_19	-840.022001598	0.165184	-839.856817598	11.7
A_DI_EE_20	-840.024375792	0.167816	-839.856559792	12.4
A_DI_EE_21	-840.022717137	0.16616	-839.856557137	12.4
A_DI_EE_22	-840.024378326	0.16796	-839.856418326	12.8
A_DI_EE_23	-840.024378331	0.167965	-839.856413331	12.8
A_DI_EE_24	-840.021634541	0.165415	-839.856219541	13.3
A_DI_EE_25	-840.022461547	0.166311	-839.856150547	13.5
A_DI_EE_26	-840.022461591	0.166363	-839.856098591	13.6
A_DI_EE_27	-840.020972994	0.165279	-839.855693994	14.7
A_DI_EE_28	-840.022419979	0.166771	-839.855648979	14.8
A_DI_EE_29	-840.022131956	0.16668	-839.855451956	15.3
A_DI_EE_30	-840.021806321	0.166545	-839.855261321	15.8
A_DI_EE_31	-840.020923613	0.165719	-839.855204613	16.0
A_DI_EE_32	-840.021756971	0.166814	-839.854942971	16.6
A_DI_EE_33	-840.021466312	0.166888	-839.854578312	17.6
A_DI_EE_34	-840.021520566	0.167082	-839.854438566	18.0
A_DI_EE_35	-840.02139604	0.16697	-839.85442604	18.0
A_DI_EE_36	-840.020853652	0.168734	-839.852119652	24.0
A_CB_cis_1	-839.996983835	0.168824	-839.828159835	0.0
A_CB_cis_2	-839.997077759	0.169218	-839.827859759	0.8

A_CB_cis_3	-839.997077738	0.169222	-839.827855738	0.8
A_CB_cis_4	-839.997316043	0.169811	-839.827505043	1.7
A_CB_cis_5	-839.996488884	0.169166	-839.827322884	2.2
A_CB_cis_6	-839.996441659	0.169238	-839.827203659	2.5
A_CB_cis_7	-839.996387797	0.169733	-839.826654797	4.0
A_CB_cis_8	-839.99623904	0.169632	-839.82660704	4.1
A_CB_cis_9	-839.995392304	0.168887	-839.826505304	4.3
A_CB_cis_10	-839.995446858	0.169175	-839.826271858	5.0
A_CB_cis_11	-839.995432086	0.169317	-839.826115086	5.4
A_CB_cis_12	-839.994892688	0.168857	-839.826035688	5.6
A_CB_cis_13	-839.995758922	0.169908	-839.825850922	6.1
A_CB_cis_14	-839.995470973	0.169652	-839.825818973	6.1
A_CB_cis_15	-839.994667015	0.169302	-839.825365015	7.3
A_CB_cis_16	-839.994661692	0.169546	-839.825115692	8.0
A_CB_cis_17	-839.994340783	0.169547	-839.824793783	8.8
A_CB_cis_18	-839.99331023	0.169249	-839.82406123	10.8
A_CB_cis_19	-839.994224026	0.170308	-839.823916026	11.1
A_CB_cis_20	-839.993510434	0.170973	-839.822537434	14.8
A_CB_cis_21	-839.992178507	0.170056	-839.822122507	15.9
A_CB_cis_22	-839.992178841	0.170068	-839.822110841	15.9
A_CB_cis_23	-839.991821841	0.169957	-839.821864841	16.5
A_CB_cis_24	-839.991101387	0.169791	-839.821310387	18.0
A_CB_cis_25	-839.990272397	0.170129	-839.820143397	21.0
A_CB_cis_26	-839.990729473	0.17087	-839.819859473	21.8
A_CB_cis_27	-839.991079658	0.171333	-839.819746658	22.1
A_TS_cis_1	-839.953150164	0.165944	-839.787206164	0.0
A_TS_cis_2	-839.954214377	0.167508	-839.786706377	1.3
A_TS_cis_3	-839.954214253	0.167512	-839.786702253	1.3
A_TS_cis_4	-839.954208954	0.167604	-839.786604954	1.6
A_TS_cis_5	-839.952807363	0.166954	-839.785853363	3.6
A_TS_cis_6	-839.953190721	0.167426	-839.785764721	3.8
A_TS_cis_7	-839.953190698	0.167426	-839.785764698	3.8
A_TS_cis_8	-839.952366528	0.166732	-839.785634528	4.1
A_TS_cis_9	-839.952366256	0.166741	-839.785625256	4.2
A_TS_cis_10	-839.952365679	0.166742	-839.785623679	4.2
A_TS_cis_11	-839.952514203	0.166995	-839.785519203	4.4
A_TS_cis_12	-839.952781362	0.167626	-839.785155362	5.4
A_TS_cis_13	-839.952781487	0.167631	-839.785150487	5.4
A_TS_cis_14	-839.952781783	0.167649	-839.785132783	5.4
A_TS_cis_15	-839.953037196	0.167935	-839.785102196	5.5
A_TS_cis_16	-839.953037219	0.167954	-839.785083219	5.6
A_TS_cis_17	-839.952503137	0.167489	-839.785014137	5.8
A_TS_cis_18	-839.952502877	0.167494	-839.785008877	5.8
A_TS_cis_19	-839.952795335	0.167906	-839.784889335	6.1
A_TS_cis_20	-839.952429471	0.167653	-839.784776471	6.4
A_TS_cis_21	-839.952429634	0.167662	-839.784767634	6.4

A_TS_cis_22	-839.952376336	0.167612	-839.784764336	6.4
A_TS_cis_23	-839.952077945	0.167631	-839.784446945	7.2
A_TS_cis_24	-839.952077952	0.167634	-839.784443952	7.3
A_TS_cis_25	-839.950939628	0.166592	-839.784347628	7.5
A_TS_cis_26	-839.952269583	0.168181	-839.784088583	8.2
A_TS_cis_27	-839.950751686	0.167257	-839.783494686	9.7
A_TS_cis_28	-839.950925678	0.167533	-839.783392678	10.0
A_TS_cis_29	-839.950864008	0.167476	-839.783388008	10.0
A_TS_cis_30	-839.951283781	0.168191	-839.783092781	10.8
A_TS_cis_31	-839.951283782	0.168192	-839.783091782	10.8
A_TS_cis_32	-839.952050625	0.169154	-839.782896625	11.3
A_TS_cis_33	-839.949976222	0.167556	-839.782420222	12.6
A_TS_cis_34	-839.949975864	0.167558	-839.782417864	12.6
A_TS_cis_35	-839.949975757	0.167569	-839.782406757	12.6
A_TS_cis_36	-839.950603599	0.168643	-839.781960599	13.8
A_TS_cis_37	-839.950437095	0.168621	-839.781816095	14.2
A_TS_cis_38	-839.950438105	0.168634	-839.781804105	14.2
A_TS_cis_39	-839.948526001	0.167472	-839.781054001	16.2
A_TS_cis_inward_1	-839.948497285	0.167833	-839.780664285	17.2
A_TS_cis_inward_2	-839.948497281	0.167835	-839.780662281	17.2
A_TS_cis_inward_3	-839.94849728	0.167835	-839.78066228	17.2
A_TS_cis_inward_4	-839.947388645	0.167255	-839.780133645	18.6
A_TS_cis_inward_5	-839.947388645	0.167255	-839.780133645	18.6
A_TS_cis_inward_6	-839.947388645	0.167255	-839.780133645	18.6
A_TS_cis_inward_7	-839.946273629	0.167751	-839.778522629	22.8
A_DI_ZE_1	-840.02511006	0.166764	-839.85834606	0.0
A_DI_ZE_2	-840.025197852	0.167034	-839.858163852	0.5
A_DI_ZE_3	-840.025173775	0.167743	-839.857430775	2.4
A_DI_ZE_4	-840.023346093	0.166391	-839.856955093	3.7
A_DI_ZE_5	-840.023504521	0.166591	-839.856913521	3.8
A_DI_ZE_6	-840.023504521	0.166591	-839.856913521	3.8
A_DI_ZE_7	-840.02373257	0.167021	-839.85671157	4.3
A_DI_ZE_8	-840.023418019	0.166727	-839.856691019	4.3
A_DI_ZE_9	-840.022440479	0.166265	-839.856175479	5.7
A_DI_ZE_10	-840.022270378	0.166269	-839.856001378	6.2
A_DI_ZE_11	-840.022479551	0.166529	-839.855950551	6.3
A_DI_ZE_12	-840.023406517	0.167621	-839.855785517	6.7
A_DI_ZE_13	-840.023406639	0.167641	-839.855765639	6.8
A_DI_ZE_14	-840.024004096	0.168823	-839.855181096	8.3
A_DI_ZE_15	-840.02099283	0.166592	-839.85440083	10.4
A_DI_ZE_16	-840.020688228	0.166297	-839.854391228	10.4
A_DI_ZE_17	-840.019884305	0.165917	-839.853967305	11.5
A_DI_ZE_18	-840.019884295	0.165918	-839.853966295	11.5
A_DI_ZE_19	-840.022296918	0.168425	-839.853871918	11.7
A_DI_ZE_20	-840.022296902	0.168426	-839.853870902	11.7

A_DI_ZE_21	-840.023036389	0.169202	-839.853834389	11.8
A_DI_ZE_22	-840.020737519	0.167381	-839.853356519	13.1
A_DI_ZE_23	-840.020737556	0.167386	-839.853351556	13.1
A_DI_ZE_24	-840.015690594	0.166212	-839.849478594	23.3
A_DI_ZE_25	-840.015877726	0.167038	-839.848839726	25.0
A_DI_ZE_26	-840.01486501	0.166502	-839.84836301	26.2
A_DI_ZE_27	-840.01525906	0.1669	-839.84835906	26.2
A_DI_ZE_28	-840.014865124	0.166522	-839.848343124	26.3
A_DI_ZE_29	-840.014454971	0.166194	-839.848260971	26.5
A_DI_ZE_30	-840.014720423	0.166828	-839.847892423	27.4
B_CB_trans_1	-420.880372622	0.076292	-420.804080622	0.0
B_CB_trans_2	-420.880223321	0.076488	-420.803735321	0.9
B_CB_trans_3	-420.879629471	0.075895	-420.803734471	0.9
B_TS_trans_1	-420.845093153	0.07455	-420.770543153	0.0
B_TS_trans_2	-420.844163728	0.0744	-420.769763728	2.0
B_TS_trans_inward_1	-420.831360041	0.074405	-420.756955041	35.7
B_DI_EE_1	-420.91893502	0.075325	-420.84361002	0.0
B_DI_EE_2	-420.9183643	0.075627	-420.8427373	2.3
B_DI_EE_3	-420.912942424	0.074717	-420.838225424	14.1
B_DI_EE_4	-420.912326297	0.074919	-420.837407297	16.3
B_CB_cis_1	-420.879825478	0.076408	-420.803417478	0.0
B_CB_cis_2	-420.879072254	0.076292	-420.802780254	1.7
B_CB_cis_3	-420.879072271	0.076296	-420.802776271	1.7
B_TS_cis_1	-420.843387623	0.074923	-420.768464623	0.0
B_TS_cis_2	-420.843237465	0.074977	-420.768260465	0.5
B_TS_cis_3	-420.843237464	0.074977	-420.768260464	0.5
B_TS_cis_inward_1	-420.834822534	0.073977	-420.760845534	20.0
B_TS_cis_inward_2	-420.834822532	0.073977	-420.760845532	20.0
B_TS_cis_inward_3	-420.834228795	0.073618	-420.760610795	20.6
B_TS_cis_inward_4	-420.834228828	0.073634	-420.760594828	20.7
B_DI_ZE_1	-420.915541675	0.07544	-420.840101675	0.0
B_DI_ZE_2	-420.913908811	0.075257	-420.838651811	3.8
B_DI_ZE_3	-420.906419975	0.074476	-420.831943975	21.4
C_CB_trans_1	-422.136695026	0.099492	-422.037203026	0.0
C_CB_trans_2	-422.135953635	0.09958	-422.036373635	2.2
C_CB_trans_3	-422.135973664	0.099667	-422.036306664	2.4
C_CB_trans_4	-422.135273057	0.099234	-422.036039057	3.1
C_CB_trans_5	-422.135772308	0.099869	-422.035903308	3.4

C_CB_trans_6	-422.135257868	0.099511	-422.035746868	3.8
C_CB_trans_7	-422.134508251	0.099067	-422.035441251	4.6
C_CB_trans_8	-422.13386017	0.099241	-422.03461917	6.8
C_CB_trans_9	-422.133805265	0.099896	-422.033909265	8.6
C_TS_trans_1	-422.103615495	0.098129	-422.005486495	0.0
C_TS_trans_2	-422.10315802	0.0978	-422.00535802	0.3
C_TS_trans_3	-422.102758547	0.097959	-422.004799547	1.8
C_TS_trans_4	-422.102331171	0.097795	-422.004536171	2.5
C_TS_trans_inward_1	-422.094003295	0.098553	-421.995450295	26.4
C_DI_EE_1	-422.178637604	0.099102	-422.079535604	0.0
C_DI_EE_2	-422.178030523	0.09921	-422.078820523	1.9
C_DI_EE_3	-422.172898443	0.098416	-422.074482443	13.3
C_DI_EE_4	-422.172918717	0.098526	-422.074392717	13.5
C_DI_EE_5	-422.172295157	0.098586	-422.073709157	15.3
C_DI_EE_6	-422.172228459	0.099916	-422.072312459	19.0
C_CB_cis_1	-422.135953939	0.099562	-422.036391939	0.0
C_CB_cis_2	-422.135342187	0.100012	-422.035330187	2.8
C_CB_cis_3	-422.135070967	0.099929	-422.035141967	3.3
C_CB_cis_4	-422.134647461	0.100038	-422.034609461	4.7
C_TS_cis_1	-422.104467339	0.099422	-422.005045339	0.0
C_TS_cis_2	-422.102633328	0.098636	-422.003997328	2.8
C_TS_cis_3	-422.102644964	0.09876	-422.003884964	3.0
C_TS_cis_4	-422.103045681	0.099345	-422.003700681	3.5
C_TS_cis_inward_1	-422.097059	0.098209	-421.99885	16.3
C_TS_cis_inward_2	-422.09628248	0.097782	-421.99850048	17.2
C_DI_ZE_1	-422.175444236	0.098944	-422.076500236	0.0
C_DI_ZE_2	-422.173743475	0.099286	-422.074457475	5.4
C_DI_ZE_3	-422.169751784	0.09992	-422.069831784	17.5
C_DI_ZE_4	-422.165956213	0.099122	-422.066834213	25.4
D_CB_trans_1	-575.858028067	0.14326	-575.714768067	0.0
D_CB_trans_2	-575.857245482	0.143361	-575.713884482	2.3
D_CB_trans_3	-575.857130352	0.14359	-575.713540352	3.2
D_TS_trans_1	-575.826806548	0.142554	-575.684252548	0.0
D_TS_trans_2	-575.825912816	0.142361	-575.683551816	1.8
D_TS_trans_inward_1	-575.81041756	0.141628	-575.66878956	40.6
D_DI_EE_1	-575.897272447	0.143047	-575.754225447	0.0

D_DI_EE_2	-575.896657254	0.142774	-575.753883254	0.9
D_DI_EE_3	-575.891117066	0.142615	-575.748502066	15.0
D_DI_EE_4	-575.890458802	0.142276	-575.748182802	15.9
D_CB_cis_1	-575.857571552	0.14425	-575.713321552	0.0
D_CB_cis_2	-575.85671654	0.143909	-575.71280754	1.3
D_CB_cis_3	-575.856717711	0.143927	-575.712790711	1.4
D_TS_cis_1	-575.826998376	0.143126	-575.683872376	0.0
D_TS_cis_2	-575.826301815	0.143321	-575.682980815	2.3
D_TS_cis_3	-575.826301433	0.143321	-575.682980433	2.3
D_TS_cis_inward_1	-575.814169862	0.141522	-575.672647862	29.5
D_TS_cis_inward_2	-575.813153114	0.141515	-575.671638114	32.1
D_DI_ZE_1	-575.894300911	0.142849	-575.751451911	0.0
D_DI_ZE_2	-575.89250547	0.142318	-575.75018747	3.3
D_DI_ZE_3	-575.884178442	0.142776	-575.741402442	26.4
E_CB_trans_1	-508.356926151	0.068004	-508.288922151	0.0
E_CB_trans_2	-508.356926141	0.068007	-508.288919141	0.0
E_CB_trans_3	-508.355265155	0.068	-508.287265155	4.4
E_CB_trans_4	-508.355263392	0.068039	-508.287224392	4.5
E_CB_trans_5	-508.355263482	0.068045	-508.287218482	4.5
E_CB_trans_6	-508.356156426	0.068998	-508.287158426	4.6
E_CB_trans_7	-508.356134256	0.06904	-508.287094256	4.8
E_CB_trans_8	-508.356134357	0.069048	-508.287086357	4.8
E_CB_trans_9	-508.355252321	0.068904	-508.286348321	6.8
E_TS_trans_1	-508.326317517	0.067286	-508.259031517	0.0
E_TS_trans_2	-508.32631754	0.06729	-508.25902754	0.0
E_TS_trans_3	-508.325424864	0.067305	-508.258119864	2.4
E_TS_trans_inward_1	-508.305112763	0.066893	-508.238219763	54.6
E_DI_EE_1	-508.395600798	0.067865	-508.327735798	0.0
E_DI_EE_2	-508.394849483	0.0676	-508.327249483	1.3
E_DI_EE_3	-508.394603115	0.068352	-508.326251115	3.9
E_DI_EE_4	-508.393901935	0.06773	-508.326171935	4.1
E_DI_EE_5	-508.389504606	0.067656	-508.321848606	15.5
E_DI_EE_6	-508.388843459	0.067912	-508.320931459	17.9
E_CB_cis_1	-508.356248879	0.069285	-508.286963879	0.0
E_CB_cis_2	-508.356247839	0.069284	-508.286963839	0.0
E_CB_cis_3	-508.356109554	0.069664	-508.286445554	1.4
E_CB_cis_4	-508.355534301	0.069513	-508.286021301	2.5
E_CB_cis_5	-508.355515836	0.069853	-508.285662836	3.4

E_CB_cis_6	-508.354456977	0.069298	-508.285158977	4.7
E_CB_cis_7	-508.354024564	0.069119	-508.284905564	5.4
E_TS_cis_1	-508.328326433	0.068738	-508.259588433	0.0
E_TS_cis_2	-508.326552942	0.068658	-508.257894942	4.4
E_TS_cis_3	-508.324964053	0.067695	-508.257269053	6.1
E_TS_cis_4	-508.32469013	0.067842	-508.25684813	7.2
E_TS_cis_inward_1	-508.308158282	0.066585	-508.241573282	47.3
E_TS_cis_inward_2	-508.307574799	0.066694	-508.240880799	49.1
E_TS_cis_inward_3	-508.307315234	0.066504	-508.240811234	49.3
E_TS_cis_inward_4	-508.307315264	0.066505	-508.240810264	49.3
E_TS_cis_inward_5	-508.306628723	0.066544	-508.240084723	51.2
E_DI_ZE_1	-508.392577936	0.067611	-508.324966936	0.0
E_DI_ZE_2	-508.3920936	0.068424	-508.3236696	3.4
E_DI_ZE_3	-508.390879348	0.067931	-508.322948348	5.3
E_DI_ZE_4	-508.384812602	0.067794	-508.317018602	20.9
F_CB_trans_1	-459.280113538	0.0984	-459.181713538	0.0
F_CB_trans_3	-459.279552561	0.098285	-459.181267561	1.2
F_CB_trans_4	-459.278029907	0.098615	-459.179414907	6.0
F_CB_trans_5	-459.278404491	0.099186	-459.179218491	6.6
F_CB_trans_6	-459.277371887	0.098705	-459.178666887	8.0
F_CB_trans_7	-459.277174645	0.098836	-459.178338645	8.9
F_TS_trans_1	-459.25484834	0.097265	-459.15758334	0.0
F_TS_trans_2	-459.253675263	0.096989	-459.156686263	2.4
F_TS_trans_3	-459.253976373	0.097349	-459.156627373	2.5
F_TS_trans_4	-459.25274091	0.097506	-459.15523491	6.2
F_TS_trans_inward_1	-459.233727585	0.097648	-459.136079585	56.5
F_TS_trans_inward_2	-459.233727652	0.09765	-459.136077652	56.5
F_TS_trans_inward_3	-459.23316012	0.09746	-459.13570012	57.5
F_DI_EE_1	-459.320429832	0.09899	-459.221439832	0.0
F_DI_EE_2	-459.319738462	0.098887	-459.220851462	1.5
F_DI_EE_3	-459.318876789	0.098644	-459.220232789	3.2
F_DI_EE_4	-459.318065815	0.098344	-459.219721815	4.5
F_DI_EE_5	-459.313319763	0.097939	-459.215380763	15.9
F_DI_EE_6	-459.314129645	0.098827	-459.215302645	16.1
F_DI_EE_7	-459.312488866	0.097738	-459.214750866	17.6
F_CB_cis_1	-459.278133842	0.098826	-459.179307842	0.0
F_CB_cis_2	-459.278469847	0.099457	-459.179012847	0.8
F_CB_cis_3	-459.27728411	0.09931	-459.17797411	3.5
F_CB_cis_4	-459.276479949	0.099108	-459.177371949	5.1

F_CB_cis_5	-459.275551018	0.098964	-459.176587018	7.1
F_CB_cis_6	-459.274709067	0.098603	-459.176106067	8.4
F_TS_cis_1	-459.253579742	0.097656	-459.155923742	0.0
F_TS_cis_2	-459.252954153	0.097812	-459.155142153	2.1
F_TS_cis_3	-459.253144048	0.098636	-459.154508048	3.7
F_TS_cis_4	-459.251414895	0.098764	-459.152650895	8.6
F_TS_cis_inward_1	-459.235452914	0.097254	-459.138198914	46.5
F_TS_cis_inward_2	-459.234487706	0.097229	-459.137258706	49.0
F_TS_cis_inward_3	-459.233136265	0.096397	-459.136739265	50.4
F_TS_cis_inward_4	-459.233136264	0.096397	-459.136739264	50.4
F_TS_cis_inward_5	-459.232165005	0.09629	-459.135875005	52.6
F_DI_ZE_1	-459.318357869	0.09931	-459.219047869	0.0
F_DI_ZE_2	-459.315927416	0.097386	-459.218541416	1.3
F_DI_ZE_3	-459.31628657	0.09938	-459.21690657	5.6
F_DI_ZE_4	-459.314227821	0.09804	-459.216187821	7.5

Structural parameters of the investigated cyclobutenes

Table S2. Bond lengths C_1-C_2 (Å) for the cyclobutenes **A-F**

System	$r(C_1\text{-}C_2)$, Å	
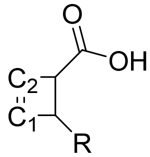	*Trans*	*Cis*
**A:** $CH(COOMe)_2$	1.573	1.585
**B:** C≡CH	1.600	1.603
**C:** $CH{=}CH_2$	1.601	1.604
**D:** Ph	1.606	1.610
**E:** N_3	1.595	1.592
**F:** OMe	1.605	1.607

Outward vs inward rotation and calculated half-lives

Table S3. Calculated $\Delta G_{298}^{‡}$ activation barriers of outward and inward rotations of the investigated *trans*- and *cis*-cyclobutenes and estimated half-lives (in brackets)

System	*Trans*		*Cis*	
	Outward ($t_{1/2}$)	Inward ($t_{1/2}$)	Outward ($t_{1/2}$)	Inward ($t_{1/2}$)
**A:** $CH(COOMe)_2$	25.5 (6.5 d)[a]	32.9 (4791 a)	25.7 (9.2 d)	29.8 (25.5 a)
**B:** C≡CH	21.0 (4.7 min)	29.6 (18 a)	21.9 (21.5 min)	26.7 (49.6 d)
**C:** $CH{=}CH_2$	19.9 (44.1 s)	26.2 (21.3 d)	19.7 (31.4 s)	23.6 (6.3 h)
**D:** Ph	19.1 (11.4 s)	28.9 (5.6 a)	18.5 (4.1 s)	25.5 (6.5 d)
**E:** N_3	18.8 (6.9 s)	31.8 (747 a)	17.2 (0.5 s)	28.5 (2.8 a)
**F:** OMe	15.1 (0.01 s)	28.6 (3.4 a)	14.7 (0.007 s)	25.8 (10.8 d)

[a]half-life is computed using Eyring equation $k = \frac{k_B}{h} e^{-\frac{\Delta G^{‡}}{RT}}$

Cartesian coordinates for the most stable ($\Delta G_{298,DCM}$) conformations as computed at the B3LYP-D3-SMD/def2-TZVP level of theory

A_CB_trans_1

C	-0.70730	-2.27877	-0.29450
C	-1.86651	-2.07459	-0.91869
C	-0.56337	-0.83790	0.15474
C	-1.90812	-0.58150	-0.61924
H	-2.56408	-2.70812	-1.45034
H	-0.08216	-3.14857	-0.14071
H	-1.79900	0.06255	-1.49118
C	-3.05981	-0.11603	0.23248
O	-3.29457	-0.49760	1.35292
O	-3.84324	0.77197	-0.41188
H	-4.57155	1.01692	0.18575
C	0.64345	-0.05003	-0.35812
H	0.73601	-0.17016	-1.43897
H	-0.67016	-0.68910	1.22884
C	1.90771	-0.57570	0.30499
C	0.49417	1.43630	-0.06338
O	-0.43945	1.93546	0.51280
O	2.19905	-0.36646	1.45758
O	1.53472	2.12013	-0.54951
O	2.62589	-1.33164	-0.52970
C	3.81567	-1.93800	0.02214
H	4.50757	-1.17068	0.36844
H	3.55607	-2.59839	0.84934
H	4.25712	-2.50631	-0.79211
C	1.53275	3.54601	-0.32033
H	2.45112	3.91431	-0.76977
H	0.66700	4.00641	-0.79614
H	1.52099	3.75749	0.74871

A_TS_trans_1

C	1.21005	1.42219	1.57430
C	2.37632	1.33341	0.87754
C	0.50419	0.21605	1.25934
C	2.12372	0.34719	-0.13442
H	3.31799	1.84171	1.04560
H	0.82213	2.23665	2.17619
H	1.35714	0.53650	-0.87145
C	3.14418	-0.63755	-0.51883
O	4.21589	-0.78483	0.03178
O	2.75873	-1.40088	-1.56790
H	3.46334	-2.04911	-1.73721
C	-0.98607	0.17017	1.05498
H	-1.45520	0.34051	2.03322
H	0.94187	-0.72869	1.54678

C	-1.47208	-1.20740	0.60998
C	-1.48675	1.28961	0.14900
O	-0.86718	-2.23265	0.79325
O	-2.19515	2.19496	0.50947
O	-2.67593	-1.12845	0.04170
O	-1.00803	1.14974	-1.09124
C	-1.40524	2.15066	-2.05310
H	-2.48993	2.16726	-2.15474
H	-1.04843	3.13368	-1.74677
H	-0.94061	1.85396	-2.98951
C	-3.26457	-2.37190	-0.40163
H	-2.63060	-2.84171	-1.15310
H	-3.40148	-3.04754	0.44206
H	-4.22534	-2.10091	-0.83047

A_DI_EE_1

C	-1.21938	-0.20862	-0.56435
C	-2.50448	-0.15515	0.08982
C	-0.06491	-0.18871	0.11194
C	-3.67938	-0.17036	-0.55865
H	-2.51068	-0.09716	1.17441
H	-1.20997	-0.26598	-1.64930
H	-3.72880	-0.22790	-1.63894
C	-4.95110	-0.10501	0.17252
O	-5.08721	-0.02792	1.37494
O	-6.00983	-0.13999	-0.67100
H	-6.81785	-0.09555	-0.13191
C	1.28037	-0.20396	-0.54891
H	1.18833	-0.45049	-1.60675
H	-0.06747	-0.13437	1.19472
C	2.22558	-1.21940	0.09023
C	1.89584	1.19851	-0.49131
O	1.97068	1.94582	-1.43205
O	1.94734	-1.94459	1.01064
O	2.30144	1.48928	0.74790
O	3.40962	-1.19822	-0.52901
C	2.88931	2.79460	0.94467
H	2.17431	3.57516	0.68653
H	3.14074	2.84373	2.00064
H	3.78601	2.89931	0.33437
C	4.42121	-2.09532	-0.02087
H	4.63828	-1.86950	1.02301
H	4.09250	-3.13022	-0.11245
H	5.29983	-1.92167	-0.63624

A_CB_cis_1

C	-0.80745	-2.50120	0.05799
C	-2.12539	-2.32037	-0.00678

C	-0.46947	-1.14465	-0.52784
C	-2.03729	-0.92989	-0.61893
H	-2.98673	-2.92725	0.23908
H	-0.17826	-3.32115	0.37875
H	-2.38810	-0.86611	-1.64799
C	-2.65744	0.17960	0.18845
O	-2.37926	0.46344	1.33022
O	-3.61582	0.82778	-0.50061
H	-3.99045	1.51300	0.08070
C	0.36615	-0.20301	0.34222
H	-0.04090	-0.16176	1.35041
H	-0.03439	-1.18291	-1.52801
C	0.37374	1.21617	-0.20506
C	1.78490	-0.74056	0.47085
O	2.23549	-1.25318	1.46429
O	-0.21494	1.58221	-1.19208
O	2.45887	-0.60185	-0.67767
O	1.10869	2.01557	0.57412
C	1.21074	3.39730	0.16990
H	1.69670	3.47273	-0.80295
H	0.22294	3.85489	0.12255
H	1.81542	3.88048	0.93274
C	3.81317	-1.10044	-0.69747
H	4.42072	-0.57835	0.04145
H	4.18209	-0.90223	-1.70021
H	3.82682	-2.17081	-0.49268

A_TS_cis_1

C	-1.10901	-2.30947	-0.05303
C	-2.44655	-2.26836	-0.30199
C	-0.57999	-1.08903	-0.57569
C	-2.76233	-0.89326	-0.52864
H	-3.15372	-3.08755	-0.36229
H	-0.53057	-3.05432	0.48108
H	-3.45293	-0.58981	-1.31276
C	-2.58897	0.12894	0.51554
O	-2.27374	-0.02650	1.67827
O	-2.88133	1.36544	0.02395
H	-2.74871	1.99686	0.74982
C	0.48184	-0.28420	0.11455
H	0.24259	-0.18263	1.17494
H	-0.69433	-0.89448	-1.63201
C	0.57862	1.10645	-0.50949
C	1.86007	-0.95383	0.02255
O	0.76858	1.29277	-1.68606
O	2.08878	-1.99744	-0.53083
O	0.41736	2.06705	0.39752
O	2.77110	-0.20869	0.65165

C	4.13209	-0.69658	0.62670
H	4.71638	0.04920	1.15886
H	4.19872	-1.66074	1.12989
H	4.48073	-0.79068	-0.40125
C	0.48652	3.42546	-0.09063
H	-0.29375	3.60193	-0.83057
H	0.33228	4.05510	0.78133
H	1.46361	3.61934	-0.53241

A_DI_ZE_1

C	1.36115	-0.24027	0.30919
C	2.48877	-0.43634	1.19259
C	0.10235	-0.33473	0.75414
C	3.80028	-0.38109	0.89196
H	2.23731	-0.65912	2.22594
H	1.55689	-0.01806	-0.73126
H	4.52412	-0.55782	1.67643
C	4.36569	-0.09505	-0.43321
O	3.76540	0.13879	-1.46155
O	5.72061	-0.11860	-0.39203
H	6.04401	0.07609	-1.28808
C	-1.09840	-0.12783	-0.11902
H	-0.80237	-0.02346	-1.16252
H	-0.09900	-0.55785	1.79613
C	-1.81786	1.15189	0.31866
C	-2.08752	-1.28866	-0.01970
O	-1.98264	-2.23056	0.72289
O	-2.42163	1.25758	1.35777
O	-3.09193	-1.10940	-0.88209
O	-1.66251	2.12817	-0.57408
C	-4.12404	-2.12052	-0.88813
H	-4.59124	-2.19015	0.09386
H	-4.84766	-1.79427	-1.63017
H	-3.70473	-3.08730	-1.16574
C	-2.27253	3.39805	-0.24626
H	-2.04175	4.05476	-1.08062
H	-3.35074	3.28259	-0.13952
H	-1.85053	3.79405	0.67707

B_CB_trans_1

C	0.92353	1.70652	-0.26492
C	-0.10687	1.70032	0.57757
C	0.90123	0.20273	-0.51289
C	-0.28702	0.20165	0.55810
H	-0.64742	2.47147	1.10847
H	1.57309	2.47154	-0.66802
H	-0.00605	-0.31944	1.47305
C	-1.58630	-0.34583	0.03476

O	-2.46523	0.30115	-0.48038
O	-1.65259	-1.68447	0.18114
H	-2.48977	-1.99100	-0.20929
H	0.54980	-0.06445	-1.51338
C	2.08494	-0.56665	-0.18197
C	3.07300	-1.19113	0.09874
H	3.94774	-1.74730	0.34669

B_TS_trans_1

C	-1.10132	1.56663	-0.03211
C	0.19839	1.57305	-0.41150
C	-1.32955	0.23489	0.49099
C	0.56404	0.18191	-0.50261
H	0.85996	2.41287	-0.58483
H	-1.86094	2.33363	-0.12440
H	0.08251	-0.43849	-1.24514
C	1.85898	-0.30605	-0.00950
O	2.67305	0.36462	0.59123
O	2.05346	-1.61828	-0.27519
H	2.90824	-1.87563	0.11056
H	-0.78920	-0.05266	1.38383
C	-2.49506	-0.51643	0.21801
C	-3.47387	-1.16860	-0.05104
H	-4.34229	-1.74277	-0.28174

B_DI_EE_1

C	1.20596	-0.27871	-0.00002
C	-0.13628	0.23616	-0.00001
C	2.28746	0.52761	-0.00001
C	-1.23373	-0.53904	-0.00001
H	-0.26117	1.31515	0.00000
H	1.33754	-1.35584	-0.00002
H	-1.16231	-1.61957	-0.00002
C	-2.57902	0.04999	0.00000
O	-2.84542	1.23286	0.00002
O	-3.53786	-0.90547	0.00001
H	-4.40121	-0.45804	0.00002
H	2.15038	1.60553	-0.00001
C	3.62169	0.06213	-0.00003
C	4.77046	-0.30347	-0.00001
H	5.78378	-0.63441	0.00034

B_CB_cis_1

C	-1.76690	-1.13218	-0.20960
C	-0.63916	-1.83797	-0.24783
C	-1.19195	-0.00386	0.63899
C	0.15799	-0.86691	0.58823
H	-0.36152	-2.78502	-0.68932

H	-2.76407	-1.26912	-0.60552
H	0.45009	-1.21602	1.58017
C	1.32689	-0.18213	-0.06412
O	1.64795	-0.28590	-1.22204
O	1.98861	0.59813	0.81346
H	2.70207	1.05463	0.33457
H	-1.59378	0.04554	1.65383
C	-1.16307	1.32250	0.05355
C	-1.12798	2.41521	-0.44541
H	-1.10019	3.38420	-0.88797

B_TS_cis_1

C	0.61518	1.74941	-0.31615
C	-0.71889	1.89788	-0.14861
C	1.00436	0.68785	0.58968
C	-1.17172	0.70022	0.50323
H	-1.34544	2.74567	-0.40014
H	1.29230	2.26127	-0.98949
H	-1.86921	0.74345	1.33723
C	-1.16115	-0.60618	-0.17480
O	-0.86923	-0.84617	-1.32661
O	-1.58186	-1.58398	0.67217
H	-1.56984	-2.41732	0.17335
H	0.86772	0.85863	1.64985
C	1.92483	-0.32424	0.24942
C	2.65955	-1.22712	-0.06806
H	3.32028	-2.01728	-0.34349

B_DI_ZE_1

C	-0.85576	0.29682	-0.00006
C	0.22927	1.24410	0.00003
C	-2.14147	0.70727	0.00008
C	1.55273	0.98729	-0.00011
H	-0.06203	2.29101	0.00021
H	-0.62327	-0.75865	-0.00026
H	2.24604	1.81784	-0.00001
C	2.16908	-0.34658	-0.00043
O	1.60767	-1.42192	-0.00002
O	3.52098	-0.24867	0.00026
H	3.88077	-1.15200	0.00055
H	-2.36828	1.77047	0.00027
C	-3.24896	-0.17043	-0.00003
C	-4.21224	-0.89518	-0.00008
H	-5.05835	-1.54371	0.00085

C_CB_trans_1

C	0.73421	1.80592	-0.32013
C	-0.37664	1.82179	0.41538

C	0.87767	0.29088	-0.32927
C	-0.42839	0.32409	0.59579
H	-1.03035	2.60444	0.77627
H	1.35029	2.57470	-0.76928
H	-0.22488	-0.04106	1.60333
C	-1.59495	-0.41785	0.01288
O	-2.43947	0.04789	-0.71400
O	-1.58014	-1.72045	0.36860
H	-2.32674	-2.16118	-0.07315
C	2.09958	-0.29875	0.28745
H	0.68024	-0.16259	-1.30420
C	2.85373	-1.23545	-0.27818
H	2.36693	0.09219	1.26683
H	3.73700	-1.62530	0.21403
H	2.61312	-1.64446	-1.25425

C_TS_trans_1

C	1.13992	1.40431	-0.10262
C	-0.11456	1.50042	0.39311
C	1.22482	0.07672	-0.68280
C	-0.59585	0.14060	0.45907
H	-0.68142	2.38452	0.66038
H	1.95717	2.11556	-0.06391
H	-0.11020	-0.55466	1.12900
C	-1.95874	-0.21020	0.05881
O	-2.75662	0.54759	-0.45796
O	-2.25619	-1.51108	0.30183
H	-3.15923	-1.67055	-0.02038
C	2.34333	-0.82508	-0.50243
H	0.59683	-0.11162	-1.54331
C	3.25907	-0.67942	0.46474
H	2.42069	-1.66221	-1.18920
H	4.10090	-1.35500	0.55207
H	3.18983	0.11773	1.19701

C_DI_EE_1

C	-1.04652	-0.49768	-0.00001
C	0.25196	0.11428	-0.00003
C	-2.19269	0.21618	-0.00001
C	1.41435	-0.56451	-0.00004
H	0.29087	1.20002	-0.00004
H	-1.09296	-1.58341	0.00000
H	1.43582	-1.64725	-0.00003
C	2.70142	0.13507	-0.00007
O	2.87109	1.33710	0.00003
O	3.74109	-0.73474	0.00005
H	4.56042	-0.21175	0.00012
C	-3.51181	-0.37056	0.00002

H	-2.13616	1.30220	-0.00002
C	-4.64204	0.34662	0.00002
H	-3.56541	-1.45602	0.00003
H	-5.61406	-0.13033	0.00003
H	-4.62402	1.43128	0.00001

C_CB_cis_1

C	-0.90959	1.87389	0.15261
C	0.40979	1.96903	-0.00669
C	-1.02948	0.48158	-0.45026
C	0.55385	0.62074	-0.66863
H	1.14251	2.73145	0.22098
H	-1.66745	2.53179	0.55901
H	0.80905	0.63856	-1.73039
C	1.39958	-0.41165	0.01885
O	1.93332	-0.28964	1.09522
O	1.48948	-1.54235	-0.71200
H	2.01200	-2.18737	-0.20442
C	-1.50506	-0.60204	0.45840
H	-1.56411	0.44248	-1.40202
C	-2.47271	-1.46273	0.16098
H	-1.01963	-0.65579	1.43014
H	-2.78993	-2.22249	0.86561
H	-2.98310	-1.43555	-0.79631

C_TS_cis_1

C	-1.00204	1.51443	0.25387
C	0.26982	1.95734	0.34232
C	-1.01742	0.51166	-0.79865
C	1.08038	0.98096	-0.34169
H	0.64082	2.87011	0.79512
H	-1.87257	1.80171	0.83314
H	1.85059	1.28051	-1.04881
C	1.26487	-0.36500	0.19219
O	0.86794	-0.81002	1.25248
O	2.00156	-1.13499	-0.66018
H	2.10032	-2.00350	-0.23811
C	-1.68962	-0.75769	-0.70829
H	-0.78844	0.86459	-1.79560
C	-2.02089	-1.31750	0.46563
H	-1.88220	-1.29151	-1.63342
H	-2.52757	-2.27375	0.51193
H	-1.78753	-0.83329	1.40504

C_DI_ZE_1

C	0.71699	0.12525	-0.00005
C	-0.29166	1.14964	-0.00007
C	2.03422	0.42569	-0.00002

C	-1.63489	1.00679	-0.00006
H	0.08136	2.17055	-0.00008
H	0.40008	-0.90949	-0.00005
H	-2.25436	1.89375	-0.00005
C	-2.35928	-0.26669	-0.00003
O	-1.89216	-1.38763	-0.00016
O	-3.70098	-0.05933	0.00021
H	-4.13016	-0.93153	0.00023
C	3.08652	-0.56251	0.00003
H	2.34155	1.46935	-0.00002
C	4.38934	-0.25534	0.00009
H	2.78006	-1.60513	0.00003
H	5.15182	-1.02425	0.00012
H	4.72738	0.77546	0.00009

D_CB_trans_1

C	-0.90911	1.92442	-0.74053
C	-1.83537	1.78971	0.20761
C	-0.43524	0.48401	-0.68467
C	-1.50270	0.34803	0.50750
H	-2.58035	2.44930	0.63169
H	-0.58480	2.75035	-1.36089
H	-1.00941	0.16345	1.46181
C	-2.56306	-0.68488	0.26510
O	-3.61889	-0.49716	-0.29082
O	-2.18414	-1.90129	0.71259
H	-2.88702	-2.53585	0.48865
H	-0.74772	-0.09832	-1.55472
C	0.99643	0.17142	-0.35338
C	1.58135	-1.00953	-0.81537
C	1.76120	1.02836	0.44257
C	2.89455	-1.33071	-0.48799
H	1.00106	-1.68281	-1.43621
C	3.07466	0.71141	0.76946
H	1.32438	1.95041	0.80784
C	3.64611	-0.47049	0.30612
H	3.33119	-2.25144	-0.85571
H	3.65411	1.38889	1.38510
H	4.66986	-0.71692	0.55971

D_TS_trans_1

C	0.64924	1.89490	0.51014
C	1.88650	1.68584	0.00496
C	0.08688	0.57241	0.70696
C	1.87230	0.31729	-0.45717
H	2.73519	2.35877	-0.03287
H	0.12670	2.82566	0.69999
H	1.21671	0.04827	-1.27341

C	3.00010	-0.58285	-0.22713
O	3.98572	-0.32259	0.43619
O	2.83278	-1.79228	-0.81974
H	3.60336	-2.33882	-0.59149
H	0.56603	-0.04634	1.45486
C	-1.26505	0.18165	0.34893
C	-2.03234	0.95267	-0.53931
C	-1.82527	-0.99395	0.87617
C	-3.32626	0.57439	-0.86579
H	-1.60222	1.84481	-0.97794
C	-3.11474	-1.37548	0.54061
H	-1.23711	-1.60210	1.55362
C	-3.87024	-0.59015	-0.32849
H	-3.91014	1.18007	-1.54770
H	-3.53565	-2.28300	0.95533
H	-4.87757	-0.88859	-0.59134

D_DI_EE_1

C	-0.45878	0.21804	-0.00769
C	-1.81050	-0.26339	-0.02081
C	0.60335	-0.61347	-0.03133
C	-2.89959	0.52787	0.01544
H	-1.95819	-1.33918	-0.06022
H	-0.31826	1.29341	0.02794
H	-2.81195	1.60632	0.05866
C	-4.24968	-0.03759	0.00253
O	-4.54148	-1.21432	-0.06270
O	-5.19635	0.93100	0.07555
H	-6.06415	0.49324	0.06210
H	0.40535	-1.68202	-0.05622
C	2.01332	-0.24469	-0.01788
C	2.97657	-1.26422	0.03330
C	2.46022	1.08727	-0.05145
C	4.33331	-0.96861	0.05554
H	2.65065	-2.29760	0.05740
C	3.81416	1.38064	-0.03066
H	1.74490	1.89801	-0.09883
C	4.75781	0.35530	0.02440
H	5.05898	-1.77156	0.09636
H	4.13932	2.41341	-0.05860
H	5.81495	0.58964	0.04025

D_CB_cis_1

C	-1.47682	-2.02480	0.25365
C	-2.51499	-1.19705	0.36277
C	-0.68490	-1.14600	-0.69638
C	-1.94358	-0.15162	-0.56341
H	-3.45930	-1.24031	0.88849

H	-1.24840	-2.99933	0.66663
H	-2.46240	-0.02097	-1.51578
C	-1.64172	1.20013	0.01233
O	-1.79727	1.53291	1.16236
O	-1.14711	2.03479	-0.92542
H	-0.92526	2.87644	-0.49087
H	-0.62959	-1.54252	-1.71213
C	0.67337	-0.64891	-0.28240
C	0.99085	-0.41992	1.05933
C	1.64424	-0.38345	-1.25120
C	2.24304	0.06481	1.42036
H	0.25118	-0.61768	1.82477
C	2.89751	0.10065	-0.89322
H	1.41365	-0.55829	-2.29598
C	3.20107	0.32715	0.44572
H	2.47164	0.23559	2.46543
H	3.63743	0.29833	-1.65942
H	4.17759	0.70113	0.72809

D_TS_cis_1

C	1.38735	1.86197	0.11663
C	2.62731	1.33343	0.03291
C	0.53741	1.03555	-0.72101
C	2.45547	0.00757	-0.50575
H	3.57894	1.79381	0.27421
H	1.03966	2.71219	0.69293
H	3.08948	-0.36242	-1.30836
C	1.79973	-1.05169	0.25772
O	1.40620	-1.01608	1.40776
O	1.68050	-2.18709	-0.48995
H	1.24593	-2.84812	0.07273
H	0.76147	1.03812	-1.78018
C	-0.78857	0.58055	-0.36465
C	-1.66330	0.09581	-1.35343
C	-1.21519	0.58024	0.97360
C	-2.93168	-0.34697	-1.01771
H	-1.33546	0.07751	-2.38632
C	-2.49023	0.14453	1.30526
H	-0.53261	0.90725	1.74604
C	-3.34972	-0.31882	0.31301
H	-3.59918	-0.71383	-1.78753
H	-2.81126	0.15296	2.33942
H	-4.34193	-0.66515	0.57509

D_DI_ZE_1

C	0.70604	-0.53138	0.00000
C	1.88724	-1.34979	0.00003
C	-0.52446	-1.08682	-0.00003

C	3.18080	-0.95793	0.00010
H	1.71121	-2.42253	0.00001
H	0.83841	0.54091	0.00002
H	3.95477	-1.71385	0.00012
C	3.65273	0.42804	0.00017
O	2.98273	1.44163	0.00003
O	5.01000	0.47912	0.00006
H	5.26441	1.41723	-0.00002
H	-0.58808	-2.17232	-0.00005
C	-1.80805	-0.39707	-0.00004
C	-2.98282	-1.16593	-0.00017
C	-1.93285	1.00299	0.00007
C	-4.23443	-0.56461	-0.00018
H	-2.90592	-2.24713	-0.00025
C	-3.18267	1.60118	0.00005
H	-1.04977	1.62829	0.00018
C	-4.33940	0.82207	-0.00008
H	-5.12708	-1.17788	-0.00028
H	-3.25916	2.68155	0.00014
H	-5.31344	1.29526	-0.00009

E_CB_trans_1

C	-0.63686	1.70338	0.32982
C	0.22805	1.58237	-0.67720
C	-0.45303	0.27999	0.80989
C	0.50032	0.11156	-0.45733
H	0.63886	2.27413	-1.39954
H	-1.23765	2.51336	0.72056
H	0.07064	-0.56210	-1.19914
C	1.90736	-0.30278	-0.13183
O	2.80999	0.44810	0.14847
O	2.04657	-1.64319	-0.14892
H	2.95861	-1.85763	0.11508
H	0.07438	0.17839	1.75588
N	-1.61479	-0.61278	0.92251
N	-2.41225	-0.60352	-0.00642
N	-3.20616	-0.67405	-0.80600

E_TS_trans_1

C	-0.35671	2.00497	0.02728
C	0.94924	1.73151	-0.20200
C	-0.94763	0.71181	0.26402
C	0.98839	0.32384	-0.52118
H	1.80584	2.39055	-0.12717
H	-0.89743	2.94371	0.01868
H	0.50880	-0.01416	-1.42928
C	2.03510	-0.54932	0.00843
O	2.85582	-0.23399	0.84818

O	1.98826	-1.79942	-0.51660
H	2.68566	-2.32358	-0.08805
H	-0.68326	0.15948	1.15738
N	-2.18608	0.41181	-0.30612
N	-2.76080	-0.62041	0.06233
N	-3.36492	-1.53791	0.29473

E_DI_EE_1

C	-0.60135	-0.61232	0.00002
C	0.66602	0.05542	0.00003
C	-1.75934	0.07298	0.00001
C	1.85733	-0.57209	0.00004
H	0.65977	1.14201	0.00005
H	-0.62398	-1.69707	0.00000
H	1.92715	-1.65260	0.00003
C	3.10967	0.18484	0.00007
O	3.22483	1.39364	-0.00002
O	4.18850	-0.63666	-0.00007
H	4.98272	-0.07628	-0.00014
H	-1.78370	1.15886	0.00003
N	-2.97953	-0.59907	-0.00001
N	-4.01201	0.07389	-0.00002
N	-5.02313	0.56750	-0.00003

E_CB_cis_1

C	1.03848	-1.99281	-0.21048
C	1.98061	-1.11028	0.12202
C	-0.09970	-1.17864	0.34944
C	1.02013	-0.10725	0.71557
H	3.05929	-1.10283	0.04866
H	1.04604	-2.97034	-0.67329
H	1.10344	0.07258	1.79052
C	0.87667	1.21610	0.01691
O	1.55821	1.61697	-0.89252
O	-0.14301	1.92747	0.54149
H	-0.22028	2.76210	0.04711
H	-0.59552	-1.59586	1.22877
N	-1.09639	-0.76929	-0.65438
N	-2.13964	-0.29759	-0.21957
N	-3.13709	0.14061	0.07477

E_TS_cis_1

C	0.39445	1.75753	-0.30484
C	-0.91636	1.80585	-0.61698
C	0.45614	0.92164	0.87895
C	-1.54979	0.73336	0.11192
H	-1.42968	2.51837	-1.25292
H	1.25420	2.19783	-0.79678

H	-2.48648	0.89905	0.63790
C	-1.30343	-0.66912	-0.21151
O	-0.48912	-1.13497	-0.98764
O	-2.12822	-1.48157	0.50596
H	-1.89989	-2.39539	0.26921
H	0.05296	1.28196	1.81278
N	1.39438	-0.07240	1.09709
N	2.03375	-0.50707	0.12115
N	2.70922	-0.97268	-0.64138

E_DI_ZE_1

C	0.29948	0.10490	-0.00006
C	-0.69578	1.13810	-0.00008
C	1.60904	0.41734	-0.00007
C	-2.04044	1.00475	-0.00002
H	-0.31569	2.15623	-0.00014
H	-0.00602	-0.93209	-0.00002
H	-2.65315	1.89614	-0.00002
C	-2.76858	-0.26467	0.00009
O	-2.30337	-1.38690	-0.00009
O	-4.11017	-0.05512	0.00019
H	-4.54033	-0.92680	0.00017
H	1.95874	1.44561	-0.00010
N	2.57317	-0.58717	-0.00002
N	3.76005	-0.25466	0.00001
N	4.87284	-0.08753	0.00004

F_CB_trans_1

C	0.81458	1.90992	-0.20513
C	-0.29501	1.84256	0.53012
C	0.92007	0.42049	-0.39936
C	-0.34992	0.33206	0.57793
H	-0.96547	2.58397	0.94388
H	1.42547	2.72063	-0.57953
H	-0.06708	-0.11985	1.52809
C	-1.55900	-0.34058	0.00406
O	-2.36968	0.17873	-0.72631
O	-1.63228	-1.64195	0.35909
H	-2.40710	-2.03059	-0.08292
H	0.67669	0.07663	-1.41395
O	2.10924	-0.15606	0.05050
C	2.13681	-1.57197	-0.09025
H	3.11994	-1.90892	0.23670
H	1.98256	-1.87240	-1.13380
H	1.37141	-2.05015	0.53102

F_TS_trans_1

C	0.92191	1.76717	0.00939

C	-0.40694	1.70978	0.25755
C	1.27627	0.39745	-0.27837
C	-0.69157	0.31661	0.52334
H	-1.13458	2.51282	0.22681
H	1.61824	2.59701	0.03494
H	-0.27261	-0.13398	1.41303
C	-1.88554	-0.32428	-0.00833
O	-2.64758	0.16039	-0.82583
O	-2.07518	-1.58028	0.47954
H	-2.86739	-1.93988	0.04730
H	0.91823	-0.06062	-1.19448
O	2.37303	-0.13613	0.25131
C	2.76966	-1.41944	-0.27066
H	2.96109	-1.35448	-1.34382
H	3.68195	-1.69811	0.25109
H	1.99024	-2.15836	-0.07259

F_DI_EE_1

C	1.10173	-0.14039	0.00000
C	-0.26245	0.29308	0.00001
C	2.10303	0.76658	-0.00000
C	-1.33695	-0.52246	0.00001
H	-0.44314	1.36506	0.00000
H	1.30605	-1.20375	0.00001
H	-1.22843	-1.59976	0.00001
C	-2.69234	0.01571	0.00001
O	-3.01339	1.18861	-0.00000
O	-3.62438	-0.97391	-0.00001
H	-4.49775	-0.54812	-0.00002
H	1.89343	1.83162	-0.00000
O	3.41676	0.54097	-0.00000
C	3.87410	-0.81876	-0.00000
H	4.96025	-0.76888	-0.00001
H	3.52747	-1.34201	0.89415
H	3.52746	-1.34202	-0.89415

F_CB_cis_1

C	-0.57095	2.00570	0.24288
C	0.68129	1.90448	-0.20321
C	-1.00555	0.67306	-0.30904
C	0.52003	0.51945	-0.78921
H	1.52864	2.57749	-0.20596
H	-1.12571	2.77524	0.76384
H	0.64066	0.42380	-1.86952
C	1.22534	-0.64287	-0.14985
O	1.23941	-1.76003	-0.61241
O	1.83546	-0.33389	1.00882
H	2.22274	-1.15164	1.36698

H	-1.68262	0.73734	-1.16954
O	-1.49067	-0.23906	0.63593
C	-2.09649	-1.39336	0.05657
H	-1.40201	-1.93032	-0.59554
H	-2.99043	-1.12057	-0.51702
H	-2.38682	-2.04625	0.87920

F_TS_cis_1

C	0.39977	1.86608	-0.29093
C	-0.85289	1.89852	0.21217
C	0.99038	0.68447	0.29471
C	-1.11648	0.57807	0.73389
H	-1.53061	2.74389	0.26418
H	0.90288	2.52675	-0.98731
H	-1.58989	0.45049	1.70447
C	-1.29024	-0.56847	-0.14839
O	-1.18235	-0.62306	-1.35831
O	-1.61875	-1.68806	0.57014
H	-1.71931	-2.40721	-0.07377
H	1.18515	0.66366	1.36206
O	1.71908	-0.13524	-0.44900
C	2.45241	-1.15420	0.25954
H	1.76798	-1.77157	0.84426
H	2.94302	-1.76233	-0.49624
H	3.19929	-0.69974	0.91381

F_DI_ZE_1

C	0.78349	0.39491	-0.00000
C	-0.32019	1.30725	-0.00002
C	2.04675	0.87555	-0.00001
C	-1.64522	1.02709	-0.00002
H	-0.05677	2.36231	-0.00005
H	0.58343	-0.66499	0.00002
H	-2.35367	1.84429	-0.00005
C	-2.22154	-0.31189	0.00001
O	-1.63263	-1.37733	0.00003
O	-3.58121	-0.26317	-0.00001
H	-3.90108	-1.18057	0.00000
H	2.24759	1.94267	-0.00002
O	3.18208	0.17840	0.00001
C	3.09825	-1.25494	0.00002
H	4.12455	-1.61340	0.00003
H	2.58039	-1.61061	0.89365
H	2.58041	-1.61063	-0.89362

A_TS_trans_inward_1

C	1.08076	-2.32535	-0.20573
C	1.87367	-2.12656	0.88197

C	0.45559	-1.07539	-0.50709
C	2.11498	-0.72218	0.94964
H	2.26252	-2.85589	1.58380
H	0.98608	-3.21075	-0.82508
H	2.14353	-0.19591	1.90085
C	2.81406	0.00344	-0.12865
O	3.36045	-0.44772	-1.11293
O	2.84742	1.33726	0.14946
H	3.33999	1.76411	-0.57053
C	-0.60674	-0.45368	0.37253
H	0.35978	-0.76769	-1.54691
H	-0.56325	-0.86241	1.38096
C	-0.46325	1.05978	0.50596
C	-1.99469	-0.80133	-0.18569
O	-2.19487	-1.59582	-1.06978
O	-2.94784	-0.12801	0.46008
O	-0.37130	1.64575	1.55310
O	-0.44656	1.64261	-0.69672
C	-0.27507	3.07561	-0.71285
H	-0.24415	3.35444	-1.76271
H	-1.11397	3.56261	-0.21570
H	0.65458	3.35055	-0.21661
C	-4.31064	-0.38057	0.04999
H	-4.44261	-0.13064	-1.00229
H	-4.56708	-1.42670	0.21523
H	-4.92579	0.26312	0.67295

A_TS_cis_inward_1

C	-0.42333	-2.69112	-0.40083
C	-1.60176	-2.40774	0.21753
C	0.07228	-1.45219	-0.91922
C	-2.03137	-1.15633	-0.33437
H	-2.14274	-2.98157	0.96081
H	0.02881	-3.65707	-0.59837
H	-2.19918	-1.09001	-1.40002
C	-2.74848	-0.16296	0.47727
O	-2.90330	-0.22635	1.67897
O	-3.23404	0.85995	-0.26451
H	-3.65020	1.49496	0.34251
C	0.63866	-0.36806	-0.02875
H	0.24088	-0.44271	0.98225
H	0.50162	-1.41865	-1.92005
C	0.33745	1.01569	-0.59252
C	2.16056	-0.53693	0.07889
O	0.63660	1.36242	-1.70923
O	2.78667	-1.44222	-0.41135
O	-0.31244	1.77270	0.29003

O	2.68984	0.44665	0.80877
C	4.12171	0.41008	1.00300
H	4.35370	1.27581	1.61749
H	4.41172	-0.50706	1.51500
H	4.63528	0.47591	0.04425
C	-0.70453	3.08853	-0.15670
H	-1.31945	3.01930	-1.05251
H	-1.27227	3.51794	0.66433
H	0.17796	3.69415	-0.36281

B_TS_trans_inward_1

C	-0.70752	1.65280	0.29500
C	-0.10468	1.50697	-0.90750
C	-0.96663	0.32676	0.81174
C	0.41122	0.17300	-0.94783
H	-0.01495	2.22701	-1.71310
H	-0.91813	2.55992	0.85033
H	0.32334	-0.44122	-1.84048
C	1.46487	-0.28668	-0.02392
O	2.08880	0.36589	0.78615
O	1.72717	-1.60438	-0.23323
H	2.44177	-1.85509	0.37524
C	-1.94584	-0.54306	0.27382
H	-0.75031	0.12088	1.86135
C	-2.82153	-1.28956	-0.08896
H	-3.58888	-1.94492	-0.43078

B_TS_cis_inward_1

C	-1.79062	-1.28590	-0.09461
C	-0.51289	-1.61014	-0.40281
C	-1.74481	-0.02187	0.61030
C	0.30741	-0.81450	0.46862
H	-0.14388	-2.30790	-1.14484
H	-2.70052	-1.85638	-0.24399
H	0.21562	-0.93939	1.53819
C	1.52235	-0.14614	-0.00473
O	1.93360	-0.17737	-1.14601
O	2.13203	0.56241	0.97152
H	2.89585	1.01736	0.57729
H	-2.34372	0.10203	1.51468
C	-1.43750	1.20835	-0.01989
C	-1.24124	2.28350	-0.53125
H	-1.06461	3.22412	-0.99927

C_TS_trans_inward_1

C	-0.38433	1.73341	0.45705

C	0.37302	1.71690	-0.65960
C	-0.93848	0.40546	0.62522
C	0.61969	0.33370	-0.97281
H	0.73446	2.55556	-1.24500
H	-0.52240	2.53589	1.17404
H	0.52270	-0.03953	-1.98881
C	1.38001	-0.60008	-0.14555
O	1.53172	-1.77408	-0.43778
O	1.96450	-0.07270	0.96346
H	2.41092	-0.80852	1.41221
C	-1.98357	-0.14160	-0.21277
H	-0.87278	-0.06536	1.60765
C	-2.73083	-1.19514	0.14348
H	-2.16432	0.35938	-1.15848
H	-3.51816	-1.56628	-0.50018
H	-2.57324	-1.71289	1.08306

C_TS_cis_inward_1

C	-1.28611	1.75430	-0.03212
C	0.05437	1.80099	0.13580
C	-1.61264	0.40020	-0.43222
C	0.56629	0.64526	-0.55755
H	0.65290	2.53515	0.66259
H	-2.01234	2.55831	0.02448
H	0.40183	0.56046	-1.62296
C	1.63983	-0.17956	-0.01045
O	2.17568	-0.01387	1.06839
O	1.97251	-1.20565	-0.83113
H	2.65898	-1.72933	-0.38458
C	-1.57674	-0.72609	0.47799
H	-2.26947	0.24893	-1.29138
C	-2.22143	-1.87847	0.25186
H	-1.01415	-0.58916	1.39618
H	-2.79361	-2.04211	-0.65466
H	-2.19123	-2.68587	0.97240

D_TS_trans_inward_1

C	1.15770	1.76127	0.79208
C	1.59688	1.74080	-0.48794
C	0.42236	0.54522	1.03408
C	1.55296	0.37983	-0.93193
H	1.94006	2.57369	-1.09321
H	1.37182	2.49257	1.56504
H	1.19511	0.11871	-1.92429
C	2.35254	-0.67419	-0.30387
O	3.20375	-0.57463	0.55953
O	2.06655	-1.88327	-0.87074
H	2.64123	-2.53733	-0.44112

H	0.66548	-0.02483	1.93157
C	-0.90810	0.22858	0.51396
C	-1.66058	1.11919	-0.27040
C	-1.48527	-1.00432	0.85716
C	-2.92230	0.76840	-0.72166
H	-1.24931	2.08843	-0.51909
C	-2.74778	-1.35420	0.39844
H	-0.92738	-1.69450	1.47925
C	-3.47188	-0.47209	-0.39732
H	-3.48923	1.46998	-1.32164
H	-3.16904	-2.31520	0.66703
H	-4.46031	-0.73931	-0.74944

D_TS_cis_inward_1

C	1.27937	2.24712	0.26374
C	2.13092	1.23999	0.56581
C	0.26389	1.71363	-0.61384
C	1.92521	0.22462	-0.43366
H	2.84887	1.17967	1.37648
H	1.35590	3.29851	0.52295
H	2.08705	0.46911	-1.47390
C	1.86712	-1.18860	-0.09594
O	1.95079	-1.64575	1.02902
O	1.65438	-1.97741	-1.17922
H	1.57144	-2.89254	-0.86296
H	0.04855	2.25983	-1.53409
C	-0.83928	0.83547	-0.22056
C	-1.78432	0.46895	-1.19252
C	-1.02896	0.37756	1.09500
C	-2.86086	-0.34628	-0.87140
H	-1.66220	0.82423	-2.20916
C	-2.10678	-0.43289	1.41148
H	-0.32641	0.66503	1.86520
C	-3.02712	-0.80443	0.43169
H	-3.57346	-0.62104	-1.63946
H	-2.23631	-0.77339	2.43161
H	-3.86981	-1.43503	0.68609

E_TS_trans_inward_1

C	0.25117	1.90013	0.40266
C	1.01252	1.67207	-0.68875
C	-0.60753	0.75266	0.59096
C	1.01951	0.25816	-0.93510
H	1.56439	2.39898	-1.27646
H	0.29340	2.72447	1.10656
H	0.93912	-0.14130	-1.94200
C	1.56825	-0.68825	0.03426
O	2.10297	-0.46520	1.10500

O	1.45445	-1.96613	-0.43669
H	1.85551	-2.54636	0.23046
H	-0.70969	0.28001	1.56880
N	-1.69965	0.57964	-0.25666
N	-2.46568	-0.35986	-0.02808
N	-3.24406	-1.16747	0.07646

E_TS_cis_inward_1

C	-1.40152	-1.50643	-0.52707
C	-0.16787	-1.28713	-1.03320
C	-1.44158	-0.89499	0.79116
C	0.69720	-0.95927	0.07326
H	0.15407	-1.37827	-2.06544
H	-2.20568	-2.11760	-0.92338
H	0.80737	-1.68285	0.86881
C	1.75717	0.02905	-0.04177
O	1.99700	0.69999	-1.02963
O	2.48449	0.15041	1.09892
H	3.15256	0.83939	0.94615
H	-1.76463	-1.45260	1.66583
N	-1.65438	0.46196	1.03603
N	-1.53974	1.26610	0.10561
N	-1.47104	2.08644	-0.65887

F_TS_trans_inward_1

C	0.87762	1.45968	-0.21479
C	0.26320	1.29845	0.96959
C	0.85781	0.18876	-0.93272
C	-0.49963	0.07376	0.95515
H	0.28414	1.99668	1.80256
H	1.21516	2.37991	-0.68291
H	-0.52118	-0.57908	1.82222
C	-1.62150	-0.10568	0.05692
O	-2.07249	0.65255	-0.79001
O	-2.22519	-1.31980	0.27483
H	-2.96826	-1.36738	-0.34704
H	0.40247	0.11462	-1.91941
O	1.71510	-0.80270	-0.77845
C	2.49070	-0.90600	0.42224
H	3.19536	-1.71444	0.24666
H	3.01362	0.02773	0.63157
H	1.83020	-1.15220	1.25711

F_TS_cis_inward_1

C	1.59273	-1.33499	0.16715
C	0.30838	-1.46824	0.54249

C	1.66013	-0.24213	-0.80439
C	-0.51774	-0.74012	-0.39939
H	-0.06935	-2.05753	1.37328
H	2.43740	-1.98461	0.37645
H	-0.50437	-1.06510	-1.43162
C	-1.69963	-0.02632	0.01933
O	-2.07132	0.14288	1.17242
O	-2.41433	0.48216	-1.02655
H	-3.17258	0.95471	-0.64664
H	2.03076	-0.41786	-1.81455
O	1.80120	1.04161	-0.51889
C	1.43977	1.52398	0.78240
H	1.95582	0.96379	1.56312
H	1.73448	2.56990	0.79873
H	0.36159	1.43043	0.91981
